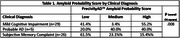# Application of a Plasma Amyloid Beta Test in Clinical Practice

**DOI:** 10.1002/alz.089646

**Published:** 2025-01-09

**Authors:** Valory Pavlik, Melissa Yu, Chi‐Ying Roy Lin, Shayla Yonce

**Affiliations:** ^1^ Baylor College of Medicine, Houston, TX USA

## Abstract

**Background:**

The gold standard Alzheimer’s disease (AD) diagnosis with amyloid PET or CSF sampling is costly and not widely available. There is growing interest in utilizing plasma biomarker tests to provide etiologic clarity in the earliest symptomatic phases. We report the uptake, and biomarker results in relation to clinical diagnosis, of a brain amyloid probability score (APS) with the Precivity AD™ test (C2N Diagnostics) offered to clinician‐selected eligible patients during their clinical workup in a memory clinic.

**Method:**

Eligibility criteria included: MMSE>=22, a CDR global score <=1.0, and a diagnosis of subjective memory complaint (SMC), mild cognitive impairment (MCI) or early AD. Consensus clinical diagnoses were assigned according to NIA‐AA criteria prior to obtaining PrecivityAD™ results. The APS, derived from a regression equation including plasma Aß_42/ 40,_ age, and APOE genotype, has a reported accuracy of 86%. To identify selection factors for referral, we used logistic regression to assess the independent association of age, sex, race/ethnicity, years of education, MMSE score, number of APOE e4 alleles, and diagnosis between participants and a sample of non‐participating patients meeting the same eligibility criteria.

**Result:**

Sixty patients were consented over 9 months. Average age was 70.8±8.06, 60.0% were male, and 11.3% persons of color. Fifty‐five percent of those with MCI received a high APS, compared to 15.4% of those with SMC. Forty‐one percent of MCI patients had a low APS compared to 61.5% with SMC (Table 1). Only a diagnosis of probable AD was associated with enrollment (less likely).

**Conclusion:**

PrecivityAD™ probability scores were generally consistent with the clinical diagnosis. An intermediate or high APS required clinician counselling regarding the implications of and indications for further testing given the clinical diagnosis. The high representation of SMC patients may reflect provider and/or patient desire for additional information on prognosis. The low usage in patients with probable AD may reflect a greater degree of provider certainty in clinical diagnosis. Longitudinal follow‐up of cognitive changes, and analysis of PET imaging results in those referred for additional testing will clarify the clinical utility of this plasma biomarker.